# Supplementary material for: Signaling cascades shape functional subpopulations of cortical astrocytes in male wild-type mice and APP/PS1dE9 Alzheimer’s disease model
Source: Nat Commun. 2026 Apr 14;17:4194. doi: 10.1038/s41467-026-71826-w (PMC13153161; doi:10.1038/s41467-026-71826-w)
Supplement: Supplementary file 1 — Supplementary Information [file 41467_2026_71826_MOESM1_ESM.pdf]

Supplementary Files

Signaling cascades shape functional subpopulations of cortical astrocytes in male wild-type mice and APP/PS1dE9 Alzheimer's disease model

Yiannis Poulot-Becq-Giraudon[1,2], Océane Guillemaud[2#], Elisa Degl'Innocenti[1#], Vivien Letenneur[2$], Karouna Bascarane[1, 2$], Tony Barbay[3$], Mie Møller Clausen[2$], Céline Derbois[4$], Martine Guillermier[1,2$], Ludmila Juricek[2], Miriam Riquelme-Perez[2,3], Tom Lakomy[1,2], Lucile Benhaim[1,2], Noëlle Dufour[2], Pauline Gipchtein[2], Fanny Petit[2], Léa Siron[1], Gwennaëlle Aurégan[2], Nathalie Dechamps[5,6], Marie-Claude Gaillard[2], Alexis-Pierre Bemelmans[2], Rémi Bos[3], Maria-Angeles Carrillo-de Sauvage[1,2], Giampaolo Milior[7], Nathalie Rouach[7], Solène Brohard[4£], Kevin Muret[4£], Eric Bonnet[4], Carole Escartin[1,2*]

Supplementary Figures

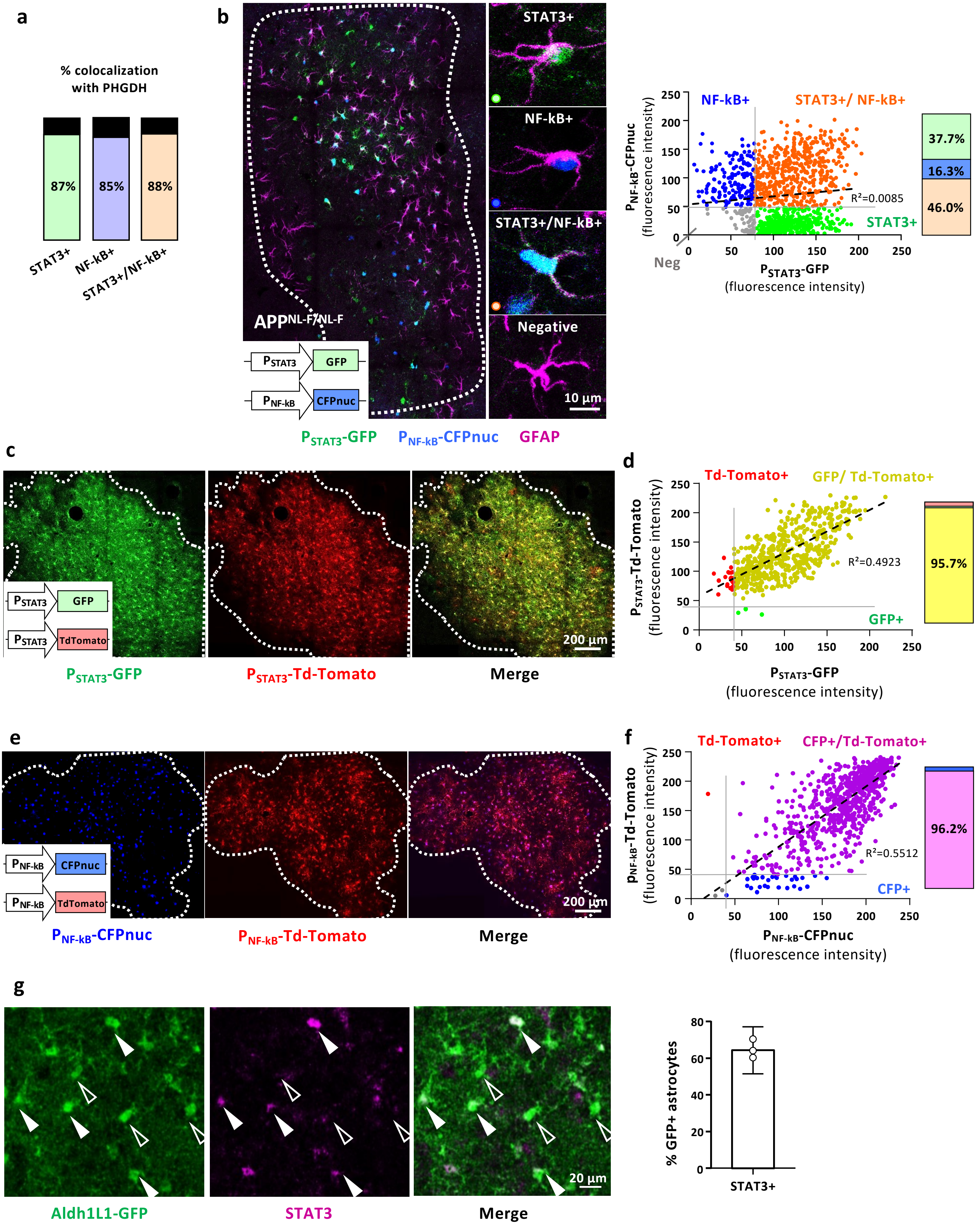

Supplementary Figure 1

Supplementary Figure 1. Additional characterization of the LV reporters

a, A similar high percentage of the three cell subpopulations co-express the astrocyte marker PHGDH. N=10. **b**, APP$^{NL-F/NL-F}$ mice were injected at 6-month-old with the two LV reporters in the PFC and analyzed 2 months later. The three astrocyte subpopulations are observed in similar proportions than those of APP/PS1 mice. Dot plot generated with all cells quantified from all mice. STAT3+ n=395; NF-kB+ n=171; STAT3+/NF-kB+ n=482; N=7. **c,** Representative image of an APP/PS1 mouse injected with LV- P_{STAT3}-GFP (green) and LV- P_{STAT3}-Td-Tomato (red) reporters in the PFC. **d,** Fluorescence intensity of GFP and Td-Tomato within single astrocytes are very well correlated, with more than 95% astrocytes co-expressing both fluorescent reporter proteins. n=541; N=3. **e,** Representative image of an APP/PS1 mouse injected with LV-P_{NF-kB}-CFPnuc (blue) and LV-P_{NF-kB}-Td-Tomato (red) reporters in the PFC. **f**, Likewise, fluorescence intensity of Td-Tomato and CFP in single astrocytes are highly correlated, with more than 96% astrocytes co-expressing both fluorescent reporter proteins. n=758; N=3. The white dashed line demarcates the transduced site displaying fluorescent astrocytes in **b, c, e**. **g,** GFP+ astrocytes in the PFC display variable STAT3 levels, with an average 65% astrocytes being STAT3+ (white arrowhead), while 35% are STAT3- (empty arrowhead), quantification on the right. N=3.

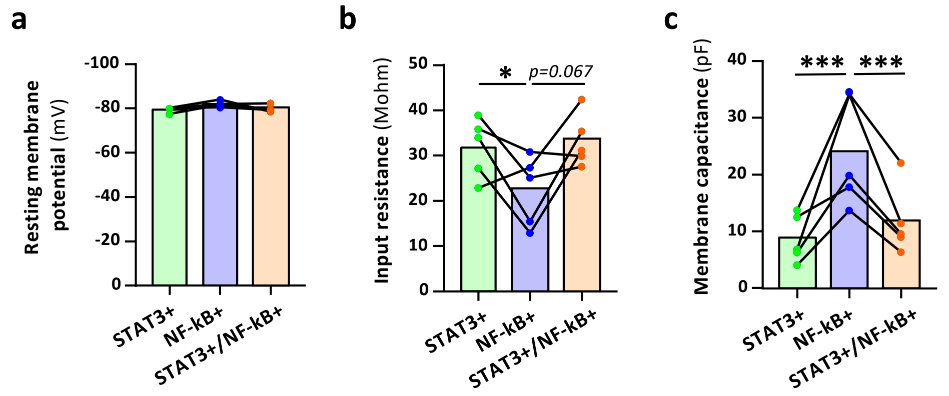

Supplementary Figure 2. Astrocyte subpopulation analysis in WT mice

a-c, Electrophysiological analysis of the three astrocyte subpopulations in WT mice. There is no significant difference in the resting membrane potential (**a**) but NF-kB+ astrocytes have a lower input resistance (**b**) and higher membrane capacitance (**c**) than the two other subpopulations. STAT3+ n=15; NF-kB+ n=9; STAT3+/NF-kB+ n=17; N=5. Linear mixed model (fixed effect: subpopulation; random effect: mouse) and Tukey's tests. In **a**: ANOVA *p*=0.080; **b.** *p*=0.038 between NF-kB+ and STAT3/NF-kB astrocytes **c.** *p*<0.001 between NF-kB+ and STAT3+ or STAT3/NF-kB astrocytes (log-transformed data). The lines connect the three astrocyte subpopulations belonging to the same mouse.

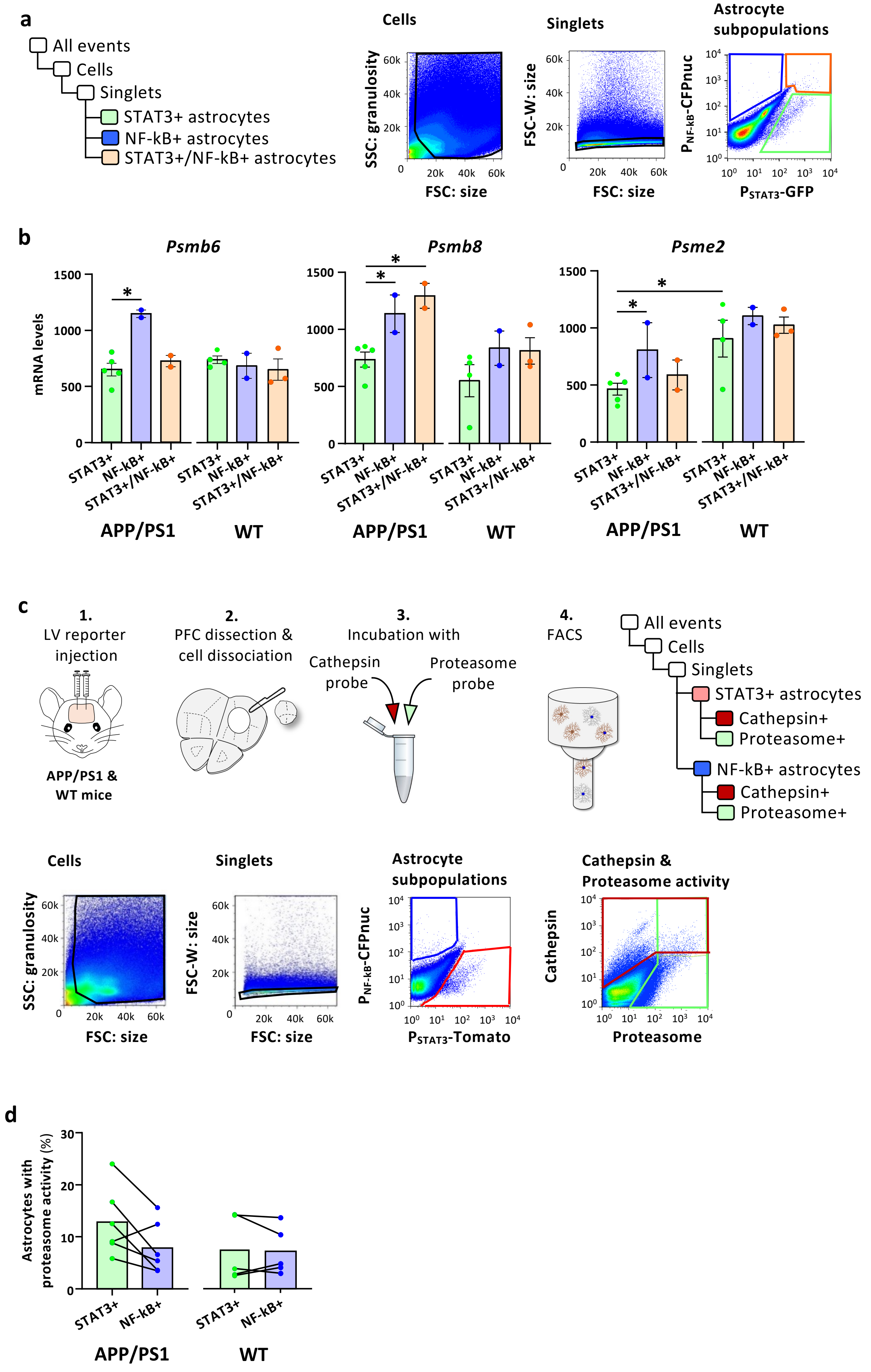

Supplementary Figure 3

Supplementary Figure 3. FACS analyses

a, Sorting strategy for the collection of STAT3+, NF-kB+ and STAT3+/NF-kB+ astrocytes from the PFC of mice injected with the reporters LV-P_{STAT3}-GFP and LV-$P_{NF\text{-}kB}$-CFPnuc, for RNA-seq analysis. **b,** Quantification of mRNA levels (TPM) for three genes encoding proteasomal subunits (*Psmb6, Psmb8,* and *Psme2*) in the three subpopulations of APP/PS1 and WT mice**.** N=2-5 per group. Statistical results are from DEseq2 analysis. **c,** Experimental design for the simultaneous assessment of proteolytic activities in single astrocytes. 1. WT and APP/PS1 mice were injected with LV-P_{STAT3}-Td-Tomato and LV-$P_{NF\text{-}kB}$-CFPnuc in the PFC. 2. PFC were collected 2 months later and dissociated. 3. Cells were incubated with fluorescent activity probes for the proteasome and cathepsins. 4. Cells were then analyzed by FACS, to measure the two fluorescent reporter proteins as well as proteasome and cathepsin activities. Sorting strategy is shown on the far right and illustrated with dotplots: cells, singlets, and CFP+ or Td-Tomato+ astrocytes were gated and the percentage of cells positive for cathepsin or proteasome were then measured. **d,** Quantification of proteasome activity in STAT3+ and NF-kB+ astrocytes in APP/PS1 and WT mice. Proteasome activity is equivalent in STAT3+ and NF-kB+ astrocytes in both genotypes. N=6 APP/PS1 and 5 WT mice. Two-way (subpopulation, genotype) ANOVA on arcsin-transformed data, $p > 0.250$ for each factor.

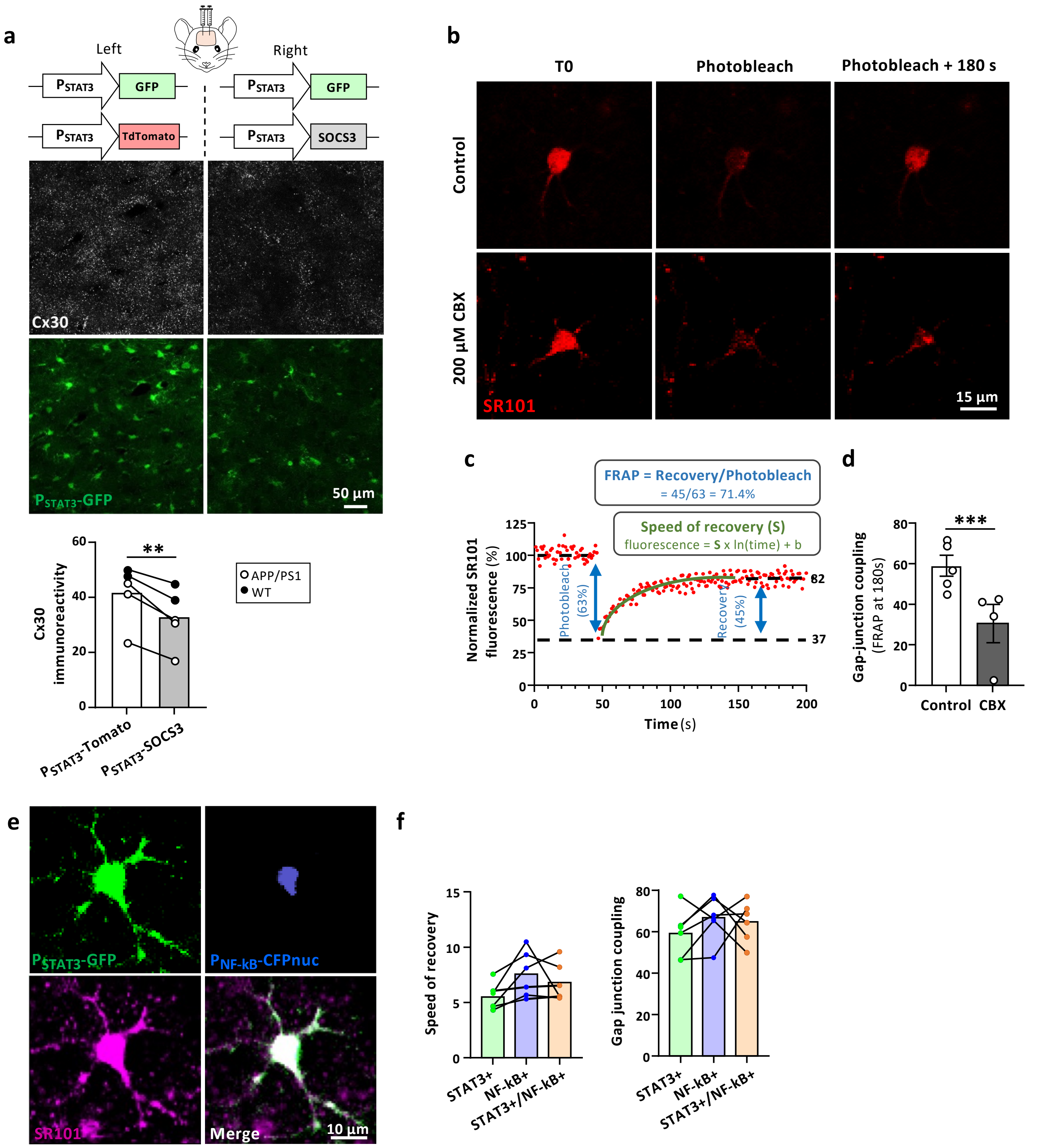

Supplementary Figure 4

Supplementary Figure 4. Quantification of connexin-mediated gap junction coupling

a, LV-P_{STAT3}-SOCS3 inhibits LV-P_{STAT3}-GFP activity (as previously quantified in **Fig. 1f**), reducing both GFP levels (green) and Cx30 (white) immunoreactivity compared to the control side injected with LV-P_{STAT3}-Td-Tomato. Paired *t* test, *p*=0.0054, N=5. Black and white circles represent WT and APP/PS1 mice. **b,** Representative two-photon images of acute PFC slices, showing SR101 labelled astrocytes (red) before (left image), 1 s after (middle image), and 180 s after photobleaching (right image) in control slices (upper panel), and slices exposed to the gap junction inhibitor carbenoxolone (200 µM CBX) during 15 min (lower panel). **c**, Representative curve for fluorescence recovery after photobleaching (FRAP) in an SR101+ astrocyte in the PFC of an APP/PS1 mouse, showing the normalized fluorescence intensity with time. The calculation used for FRAP percentage and speed of recovery are displayed on the graph. **d**, Quantification of FRAP 180 s after photobleaching in the two conditions shown in **b**. Linear mixed model (fixed effect: treatment; random effect: mouse), *p*<0.0001. Control n=36; N=5, CBX n=27; N=4. **e,** Representative two-photon images of an acute PFC slice, showing a STAT3+/NF-kB+ astrocyte also positive for SR101 (magenta). **f,** Quantification of the speed of recovery and gap junction coupling (FRAP at 180 s) in the three astrocyte subpopulations. STAT3+ n=16; NF-kB+ n=26; STAT3+/NF-kB+ n=37; N=6. Linear mixed model (fixed effect: subpopulation; random effect: mouse), *p*=0.3771 and 0.3828 respectively.

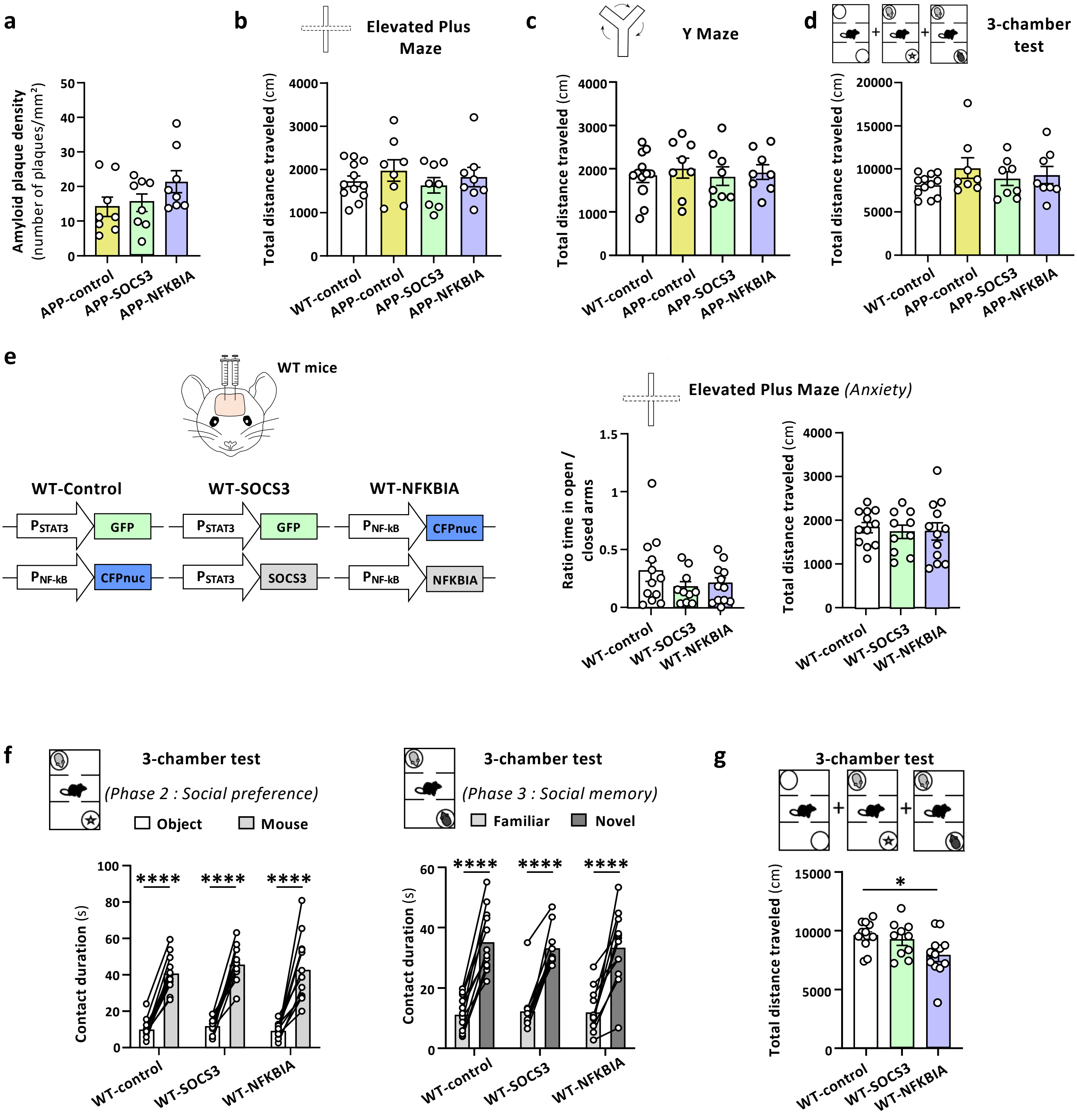

Supplementary Figure 5. Astrocyte subpopulation targeting – additional data

a, The density of BAM10-labelled amyloid plaques is similar in the three APP/PS1 groups. One-way ANOVA, p=0.178. **b-d,** Mice travelled a similar total distance at the elevated plus maze (**b**), Y maze (**c**), and the three phases of three chamber tests (**d**), in all groups. One-way ANOVA, p=0.652, 0.582, 0.393, respectively (log-transformed data). **e,** The ratio of time spent in the open arms to the closed arms is similar among the three WT groups, as well as the total distance travelled. One-way ANOVA, p=0.524 on log-transformed data and p=0.873, respectively. N=10-12/group. **f,** On the three-chamber test, mice of the three WT groups spend significantly more time with the juvenile mouse than with the object, and then with the novel mouse than the familiar one (p<0.0001). Two-way ANOVA (experimental group, stimulus) followed by Tukey's tests. **g,** The total distance travelled during the three phases of the test is significantly lower in the WT-NFKBIA group than the WT-CTR group. One-way ANOVA and Tukey test p=0.0291.

DEG considered	Upstream transcription factor	Rank	FDR	Method
**158 DEG** STAT3 < NF-kB	**NF-kB**	9	**$8.67\ 10^{-3}$**	Pscan
		2	**$7.32\ 10^{-7}$**	ChEA3-ENCODE
	**STAT3**	11	**$8.67\ 10^{-3}$**	Pscan
		4	**$2.95\ 10^{-6}$**	ChEA3-ENCODE
**477 DEG** STAT3 > NF-kB	**NF-kB**	85	$1.00\ 10^{0}$	Pscan
		33	**$6.56\ 10^{-3}$**	ChEA3-ENCODE
	**STAT3**	46	**$3.82\ 10^{-2}$**	Pscan
		56	**$1.54\ 10^{-2}$**	ChEA3-ENCODE

Supplementary Table 1. Transcription factor analysis with Pscan and ChEA3-ENCODE databases of DEG between STAT3+ and NF-kB+ astrocytes

Two methods for transcription factor analysis were applied on the list of 158 down- and 477 up-regulated genes between STAT3+ and NF-kB+ astrocytes in APP/PS1 mice. Pscan is based on DNA motif recognition in the -450 to +50 bp region of DEG promoters, while ChEA3-ENCODE inquires deposited Chip-Seq datasets for human STAT3 and RelA (NF-kB). The table provides the list of DEG considered, the transcription factor identified, its rank among 282/118 transcription factors tested by Pscan and CHE3-Encode, respectively and the FDR (in bold if below 0.05).

NF-kB was identified as an upstream regulator of the 158 down-regulated genes and STAT3 of the 477 up-regulated genes by both methods. Of note, STAT3 was also identified as a potential upstream regulator of the 158 down-regulated genes (and NF-kB of the 477 up-regulated genes by ChEA3-ENCODE), which can be linked to their known repressive activities, through chromatin remodeling or recruitment of repressors for example [1,2].

Supplementary references

1. Wingelhofer, B.*, et al.* Implications of STAT3 and STAT5 signaling on gene regulation and chromatin remodeling in hematopoietic cancer. *Leukemia* **32**, 1713-1726 (2018).
2. Arzate-Mejia, R.G., Valle-Garcia, D. & Recillas-Targa, F. Signaling epigenetics: novel insights on cell signaling and epigenetic regulation. *IUBMB life* **63**, 881-895 (2011).
