# Supplementary material for: Signaling cascades shape functional subpopulations of cortical astrocytes in male wild-type mice and APP/PS1dE9 Alzheimer’s disease model
Source: Nat Commun. 2026 Apr 14;17:4194. doi: 10.1038/s41467-026-71826-w (PMC13153161; doi:10.1038/s41467-026-71826-w)
Supplement: Supplementary file 2 — Description of Additional Supplementary Files [file 41467_2026_71826_MOESM2_ESM.pdf]

Description of Additional Supplementary Files

Supplementary Data 1. List of DEG

List of significant DEG (ordered by log2FoldChange, with adjusted *p* values from DESeq2 analysis), between the three subpopulations among APP/PS1 mice (2 orange tabs) and WT mice (3 grey tabs), as well as DEG between WT and APP/PS1 mice within the same subpopulation (2 blue tabs).

Supplementary Data 2. Pathway analysis

The five tabs display the pathways significantly enriched among the 1) up-, 2) down-regulated DEG between STAT3+ and NF-kB+ astrocytes in APP/PS1 mice, 3) 129 DEG between STAT3+ and NF-kB+ astrocytes in WT mice, and 4) up-, 5) down-regulated DEG in STAT3+ astrocytes between APP/PS1 and WT mice. Pathway analysis was performed using the databases Reactome, KEGG with an FDR set at $p < 0.05$ and GO-BP, with an FDR set at $p<0.005$ to limit the number of entries to the most significant.
