# Supplementary material for: Signaling cascades shape functional subpopulations of cortical astrocytes in male wild-type mice and APP/PS1dE9 Alzheimer’s disease model
Source: Nat Commun. 2026 Apr 14;17:4194. doi: 10.1038/s41467-026-71826-w (PMC13153161; doi:10.1038/s41467-026-71826-w)
Supplement: Supplementary file 5 — Reporting Summary [file 41467_2026_71826_MOESM5_ESM.pdf]

nature portfolio

Corresponding author(s): Carole Escartin
Last updated by author(s): Mar 20, 2026

Reporting Summary

Nature Portfolio wishes to improve the reproducibility of the work that we publish. This form provides structure for consistency and transparency in reporting. For further information on Nature Portfolio policies, see our Editorial Policies and the Editorial Policy Checklist.

Statistics

For all statistical analyses, confirm that the following items are present in the figure legend, table legend, main text, or Methods section.

n/a	Confirmed	
☐	☒	The exact sample size (*n*) for each experimental group/condition, given as a discrete number and unit of measurement
☐	☒	A statement on whether measurements were taken from distinct samples or whether the same sample was measured repeatedly
☐	☒	The statistical test(s) used AND whether they are one- or two-sided<br>*Only common tests should be described solely by name; describe more complex techniques in the Methods section.*
☐	☒	A description of all covariates tested
☐	☒	A description of any assumptions or corrections, such as tests of normality and adjustment for multiple comparisons
☐	☒	A full description of the statistical parameters including central tendency (e.g. means) or other basic estimates (e.g. regression coefficient) AND variation (e.g. standard deviation) or associated estimates of uncertainty (e.g. confidence intervals)
☐	☒	For null hypothesis testing, the test statistic (e.g. *F*, *t*, *r*) with confidence intervals, effect sizes, degrees of freedom and *P* value noted<br>*Give P values as exact values whenever suitable.*
☒	☐	For Bayesian analysis, information on the choice of priors and Markov chain Monte Carlo settings
☒	☐	For hierarchical and complex designs, identification of the appropriate level for tests and full reporting of outcomes
☐	☒	Estimates of effect sizes (e.g. Cohen's *d*, Pearson's *r*), indicating how they were calculated

Our web collection on statistics for biologists contains articles on many of the points above.

Software and code

Policy information about availability of computer code

Data collection	LAS X; Clampfit 10.7; EthoVision v17;
Data analysis	Image J; FlowJow V10; Clampfit 10.7; EthoVision v17 ; Graph Pad Prism v10; R Studio v2024.12.1;

For manuscripts utilizing custom algorithms or software that are central to the research but not yet described in published literature, software must be made available to editors and reviewers. We strongly encourage code deposition in a community repository (e.g. GitHub). See the Nature Portfolio guidelines for submitting code & software for further information.

Data

Policy information about availability of data

All manuscripts must include a data availability statement. This statement should provide the following information, where applicable:

- Accession codes, unique identifiers, or web links for publicly available datasets
- A description of any restrictions on data availability
- For clinical datasets or third party data, please ensure that the statement adheres to our policy

RNA-seq dataset is available on Gene Expression Omnibus under reference GSE290101.
All Source data are provided as a Source Data file. Raw data or images are available from the corresponding author upon request.

Research involving human participants, their data, or biological material

Policy information about studies with human participants or human data. See also policy information about sex, gender (identity/presentation), and sexual orientation and race, ethnicity and racism.

Reporting on sex and gender	N/A
Reporting on race, ethnicity, or other socially relevant groupings	N/A
Population characteristics	N/A
Recruitment	N/A
Ethics oversight	N/A

Note that full information on the approval of the study protocol must also be provided in the manuscript.

Field-specific reporting

Please select the one below that is the best fit for your research. If you are not sure, read the appropriate sections before making your selection.

- [x] Life sciences
- [ ] Behavioural & social sciences
- [ ] Ecological, evolutionary & environmental sciences

For a reference copy of the document with all sections, see nature.com/documents/nr-reporting-summary-flat.pdf

Life sciences study design

All studies must disclose on these points even when the disclosure is negative.

Sample size	Sample size were defined for each experiment based on prior experience of measurement variability and expected effect size. No formal statistical size estimation was performed.
Data exclusions	Only if a mouse brain displayed signs of local neuroinflammation due to the surgery (major increase in GFAP or Iba1 expression, excessive glial scar at the site of injection), it was excluded from the study (max. 1-2 mice per cohort). No other mice were excluded from the study.
Replication	Astrocyte subpopulations were analyzed on several independent mouse cohorts. They were found in equivalent proportions using different imaging set-ups (confocal microcopy on fixed sections, 2 photon microscopy on acute slices, electrophysiological setup).
Randomization	Mice of the appropriate genotype were randomly allocated to their group and tested in a random order.
Blinding	Behavioral tests and primary analysis were done with the experimenter blinded to the mouse group.

Reporting for specific materials, systems and methods

We require information from authors about some types of materials, experimental systems and methods used in many studies. Here, indicate whether each material, system or method listed is relevant to your study. If you are not sure if a list item applies to your research, read the appropriate section before selecting a response.

Materials & experimental systems

n/a	Involved in the study	
[ ]	[x]	Antibodies
[ ]	[x]	Eukaryotic cell lines
[x]	[ ]	Palaeontology and archaeology
[ ]	[x]	Animals and other organisms
[x]	[ ]	Clinical data
[x]	[ ]	Dual use research of concern
[x]	[ ]	Plants

Methods

n/a	Involved in the study	
[x]	[ ]	ChIP-seq
[ ]	[x]	Flow cytometry
[x]	[ ]	MRI-based neuroimaging

Antibodies

Antibodies used	mouse anti-BAM10 (Sigma, A3981, 1:500)<br>rabbit anti-Cx30 (Invitrogen, 71-2200, 1:500)<br>mouse anti-GFAP-Cy3 (Sigma, C9205, 1:1,000)<br>chicken anti-GFP (Aves labs, GFP-1020, 1:1,000)

rabbit anti-Iba1 (Wako, 019-19741, 1:1,000)
mouse anti-MOG (Millipore, MAB5680, 1:500)
chicken anti-NeuN (Sigma, ABN91, 1:1,000)
rabbit anti-PDGFRα (Cell Signaling, 3174, 1:500)
guinea pig anti-PHGDH (Frontier, 3PGDH-GP-Af198, 1:250)
rabbit anti-STAT3a (Cell Signaling, 8768P, 1:500)
goat anti-Td-Tomato (Sicgen, AB818-200, 1:1,000).

Validation — All these antibodies are standard antibodies for Neuroscience applications. They have been used and validated extensively, and they give rise to the expected staining of the targeted cell-types.

Eukaryotic cell lines

Policy information about cell lines and Sex and Gender in Research

Cell line source(s)	HEK-293 cells (ATCC® CRL-1573™) were obtained from the American Type Culture Collection (ATCC)
Authentication	The cell line was authenticated by ATCC. No additional authentication was performed after receipt
Mycoplasma contamination	ells were not tested for mycoplasma contamination after purchase
Commonly misidentified lines (See ICLAC register)	HEK-293 cells are a well-established human embryonic kidney cell line and were used as received from ATCC

Animals and other research organisms

Policy information about studies involving animals; ARRIVE guidelines recommended for reporting animal research, and Sex and Gender in Research

Laboratory animals	Experiments involve adult male APP/PS1dE9, APPNL-F/NL-F, Aldh1L1-eGFP mice and their wild-type littermates on the C57BL/6J background. (references in manuscript)
Wild animals	N/A
Reporting on sex	Only male mice were included in this study
Field-collected samples	N/A
Ethics oversight	All procedures were reviewed and approved by a local ethics committee (CETEA N°44), and by the French Ministry of Education and Research (APAFIS # #33827-2021110914102549 v4 and #4565-20 16031711426915 v3). They were performed in an authorized animal facility (#D92-032-02), in strict accordance with recommendations of the European Union (2010-63/EEC), and in compliance with the 3R guidelines.

Note that full information on the approval of the study protocol must also be provided in the manuscript.

Plants

Seed stocks	N/A
Novel plant genotypes	N/A
Authentication	N/A

Flow Cytometry

Plots

Confirm that:

- [x] The axis labels state the marker and fluorochrome used (e.g. CD4-FITC).
- [x] The axis scales are clearly visible. Include numbers along axes only for bottom left plot of group (a 'group' is an analysis of identical markers).
- [x] All plots are contour plots with outliers or pseudocolor plots.
- [x] A numerical value for number of cells or percentage (with statistics) is provided.

Methodology

Sample preparation	Twelve-month-old WT and APP mice were killed by cervical dislocation and their PFC rapidly collected in Hank's Balanced Salt Solution (HBSS) without Ca2+ and Mg2+ (Sigma). The PFC of three mice were pooled, resulting in N=5 independent PFC samples from APP mice. Cells were mechanically and enzymatically dissociated with fire-polished Pasteur pipettes and the neural tissue dissociation kit with papain (Miltenyi Biotec), following manufacturer's instructions. Myelin removal beads II (Miltenyi Biotec) were used to deplete myelin from cell suspensions using MS columns. Cells were centrifuged at 300 g for 5 min at 4°C, resuspended in 400 µl HBSS with Ca2+ and Mg2+
Instrument	Cells were sorted on a BD Influx cell sorter (BD biosciences)
Software	FACS data were analyzed with FlowJow v10.
Cell population abundance	Cell population abundances are shown in Fig 5e and Supplemental Fig. 3d
Gating strategy	For RNAseq experiments, cells were gated on a side scatter/ forward scatter plot, then singlets were selected and GFP+, CFP+ and GFP+/CFP+ astrocytes were sorted (Supplemental Fig 3a).<br>For proteostatic activity measurements, cells were gated on a side scatter/ forward scatter plot, then singlets were selected and CFP+ or Td-Tomato+ astrocytes were selected and finally the percentage of cells within each subpopulation, which had a detectable cathepsin or proteasome activity was calculated, thanks to gates defined in control samples incubated without the probe (Supplemental Fig. 3c).

☒ Tick this box to confirm that a figure exemplifying the gating strategy is provided in the Supplementary Information.
